# A comparative evaluation of sustainable asphalt binder modifiers for enhanced performance

**DOI:** 10.1038/s41598-026-46495-w

**Published:** 2026-04-13

**Authors:** Maram Saudy, Minas Guirguis, Sherif El-Badawy, Mohamed AbouZeid, Tarek Madkour

**Affiliations:** 1 The American University in Cairo, P.O. Box 74, New Cairo, 11835 Cairo Egypt; 2 Faculty of Engineering, Mansoura University, Mansoura, 35516 Egypt

**Keywords:** Sustainable binder design, Processing-property correlations, Multifunctional asphalt materials, Organic versus inorganic composites, Waste-derived polymers, Geopolymer modifiers, Environmental resilience, Chemistry, Engineering, Environmental sciences, Materials science

## Abstract

Asphalt pavement production accounts for approximately 1.5% of global carbon emissions, underscoring the need for sustainable binder modification strategies that enhance performance without compromising durability. Existing studies on eco-friendly asphalt modifiers remain largely fragmented, limiting cross-comparison and mechanistic understanding. This study develops a unified, multi-scale experimental framework to comparatively evaluate waste-derived polymers and geopolymer-based modifiers under identical processing and testing conditions. Five modifiers, crumb rubber (CR), low-density polyethylene (LDPE), a combination of CR/LDPE, fly ash (FA), and metakaolin–silica fume (MK–SF) geopolymers, were assessed alongside a commercial fiber elastomer modifier (VIATOP), using two virgin binders from Egyptian refineries to capture source dependency, resulting in twelve modified systems. Binder behavior was evaluated through integrated microstructural (SEM), chemical (FTIR and CI + SI indices), thermal and photochemical (TGA and UV–Vis), and rheological (viscosity and performance grading) analyses. Results show that geopolymer modifiers provided the most consistent improvements across scales, reducing chemical aging indices, increasing decomposition onset temperatures by approximately 10–20 °C, suppressing UV-induced aromatic growth, and maintaining workable viscosities (< 500 cP at 135 °C) with moderate Performance Grade (PG) enhancement ( ≈ + 2–4 °C). Crumb rubber offered balanced performance, yielding moderate PG increases ( ≈ + 4–5 °C) with improved aging resistance. In contrast, LDPE and VIATOP produced the largest PG increases (up to ≈ + 10–13 °C) but exhibited poorer dispersion, higher oxidation, and reduced thermal and UV stability, indicating short-term stiffening rather than long-term durability. The hybrid CR/LDPE combination showed intermediate, partially synergistic behavior. Overall, the study demonstrates that rheological gains alone are insufficient indicators of durability and highlights the decisive role of base binder chemistry. The proposed framework provides quantitative, transferable design guidance for selecting sustainable asphalt modifiers that balance stiffness, durability, and aging resistance.

## Introduction

Asphalt is one of the most extensively used construction materials worldwide, with annual production approaching 1.6 trillion tons^1,2^. The global asphalt market was valued at USD 222.4 billion in 2023 and is expected to reach USD 321.5 billion by 2028, reflecting steady growth driven primarily by roadway construction, which accounts for nearly 85% of total asphalt consumption^3,4^. Despite its economic and structural advantages, asphalt pavement production and construction contribute approximately 1.5% of global carbon emissions, corresponding to 5–10 kg of CO₂ per square meter of roadway^5,6^. These figures have intensified research efforts toward reducing the environmental footprint of asphalt pavements, particularly through sustainable binder modification strategies^7,8^.

Conventional asphalt binders exhibit inherent limitations, including temperature susceptibility, rutting, fatigue cracking, and progressive aging under oxidative and ultraviolet exposure^9–12^. These degradation mechanisms shorten service life and increase maintenance demands, motivating the development of modified binders with improved durability and performance consistency^13–15^. Recent studies have demonstrated that binder modification, rather than mixture redesign alone, offers a direct and effective pathway for enhancing pavement longevity while reducing lifecycle environmental impacts.

Among emerging strategies, waste-derived polymers have attracted attention for their potential to enhance asphalt performance. Waste polymers such as crumb rubber (CR) and low-density polyethylene (LDPE) have been reported to improve specific asphalt properties. For instance, CR improves elasticity and cracking resistance through elastomeric network formation and binder swelling. Specifically^16^, reported enhanced binder-interaction morphology^17^; optimized rubberized mixtures to improve fatigue life^18^; used microscopy to assess cracking susceptibility, and^19^reported that CR improves low-temperature rheological properties. CR also boosts deformation and aging resistance when blended with recycled polyethylene^20^. demonstrated composites with higher thermal stability and reduced aging hardening, while^21^ confirmed rheological indices such as G*sinδ enhancement for long-term deformation control.

Similarly, low-density polyethylene (LDPE) has been widely reported to enhance deformation resistance and cracking performance in asphalt mixtures^22^. demonstrated that incorporating 4–10% LDPE increased Marshall stability by approximately 30%. Likewise^23^, confirmed that a 4% LDPE content by weight of binder increased Marshall stability from 10.78 kN to 11.73 kN, reduced air voids from 4.12% to 3.97%, and raised the voids filled with asphalt from 71.5% to 72.95%, indicating improved bulk density and compaction. In addition, the tensile strength ratio (TSR) increased from 83% to 88%, with the wet indirect tensile strength exceeding 900 kPa, confirming enhanced resistance to moisture damage and overall mixture durability. Using the Multiple Stress Creep Recovery (MSCR) test^24^, demonstrated that incorporation of 3–6% LDPE significantly reduced the non-recoverable creep compliance (Jnr) to approximately 0.2 kPa⁻¹, compared with 0.5–1.0 kPa⁻¹ for the unmodified binder. Concurrently, elastic recovery increased to nearly 20% at 0.1 kPa and 30% at 3.2 kPa stress levels. These findings indicate a marked enhancement in rutting resistance, reflecting improved elastic response and reduced permanent deformation under repeated loading. Consistent with these findings^25^, observed reduced rut depth in wheel-tracking tests, while^26^reported enhanced cracking resistance. Additionally^27^, noted an increase in binder softening point to 62 °C, reflecting improved high-temperature performance.

In parallel, geopolymer modifiers synthesized from industrial byproducts, particularly fly ash (FA) and silica fume (SF), have shown considerable potential in enhancing thermal stability, aging resistance, and long-term durability of asphalt binders^28^. demonstrated that FA-based geopolymers increased binder softening point and improved rutting resistance through systematic synthesis and rheological evaluation. Similarly^29^, reported that waste-derived geopolymers reduced aging susceptibility while enhancing high-temperature performance, as evidenced by comprehensive binder property testing. Consistent with these findings^30^, further confirmed the durability improvements associated with geopolymer activation of industrial byproducts.

Commercial fiber-based elastomer modifiers (FEM), such as VIATOP, have been increasingly adopted in heavy-traffic pavement applications^31^. demonstrated that VIATOP (at 0.3–0.4% by mix weight) increased the stiffness modulus to 12,500–14,200 MPa at 5 °C (vs. 11,800 MPa for reference HMA), reduced rut depths to 1.2–1.5 mm after 10,000 wheel passes at 60 °C (vs. 2.1 mm for reference), and preserved low-temperature fracture resistance by shifting critical cracking temperatures to −22 °C (vs. −18°C for reference). Moreover^32^, demonstrated that VIATOP enhanced the Marshall stability by 15% over control, while also contributing to improved rutting resistance (rut depth = 3 mm after 10,000 cycles) and extended fatigue life with 30–40% gain. More recently^33^, reported that fiber-enhanced mixtures (FEM), specifically incorporating VIATOP, increased Marshall stability by up to 25% compared with control specimens. In addition, binder drain down was reduced by approximately 30–35%, while rutting resistance improved substantially, with rut depth decreasing by 20–30% after 5,000–10,000 loading cycles. These results confirm the contribution of fiber additives to enhancing structural stability, mitigating binder migration, and improving long-term pavement durability within sustainable asphalt systems.

Despite these advances, existing studies predominantly examine modifiers in isolation, often using different binder sources, preparation protocols, and evaluation methods^34^. For instance^35^, highlighted challenges in characterizing CR-modified asphalt based upon previous work due to their varying preparation and methodological inconsistencies. Moreover^36^, noted that inconsistencies in testing protocols across studies may lead to divergent mechanistic interpretations. For instance, TGA–FTIR analyses vary depending on testing parameters such as heating rate, atmospheric environment, and data-processing procedures. Furthermore, the review by^37^ highlighted substantial methodological variability across aging protocols applied to modified asphalt binders, including differences in conditioning procedures, aging durations, and performance characterization techniques. Such multi-dimensional inconsistencies hinder direct comparison of aging responses among modified binders. Consequently, cross-study performance trends remain difficult to interpret, and the fundamental processing–property relationships governing binder aging behavior are not yet clearly established.

Additionally, asphalt binders are inherently complex, multi-fractional systems, and their response to modification is strongly governed by binder origin, modifier chemistry, dosage, and processing route^38^. linked origin-dependent microstructural responses to variations in maltenes–asphaltenes interactions, underscoring the role of intrinsic chemical composition. While^39^demonstrated that oxidative aging behavior varies systematically with modifier dosage, highlighting the sensitivity of performance evolution to formulation parameters. Similarly^40^, reported that local binders (i.e., Egyptian binders in their study) exhibit microphase-specific rheo-mechanical responses associated with their unique chemical composition. Complementing these findings^41^, quantitatively modeled the influence of SARA fraction distribution (saturates, aromatics, resins, and asphaltenes) on rheological performance, reinforcing the compositional basis of binder behavior. Processing conditions have likewise been shown to exert a decisive influence. For instance^42^, reported that high-shear versus low-shear mixing significantly alters the dispersion of modifiers, leading to differences in rheological and mechanical performance. Moreover^43^, observed that recycled material blends respond differently depending on applied aging protocols, and^44^ confirmed that rubber modifier performance is strongly dependent on activation method. Collectively, these studies underscore the coupled influence of composition and processing on the performance and durability of modified asphalt systems.

Accordingly, the need is not for new characterization techniques, but for a structured, cross-comparative evaluation that integrates existing methods to generate actionable insights into how different modifier classes perform relative to one another under consistent conditions. The absence of a unified, cross-comparative framework integrating organic and inorganic modifiers under consistent conditions represents a critical gap in current research.

### Research gap and objective

The key research gap lies in the lack of systematic, multi-scale comparative studies that simultaneously evaluate waste-derived polymers, geopolymer modifiers, and commercial additives within a single experimental framework while accounting for binder-source dependency. Without such an approach, it remains challenging to establish transferable processing–property correlations that can guide rational binder design.

Accordingly, this study develops a unified comparative framework to evaluate eco-friendly asphalt modifiers across chemical, thermal, aging, and microstructural dimensions. Five modifiers were investigated: three waste-derived systems (CR, LDPE, and a hybrid CR/LDPE) and two geopolymer-based modifiers (FA and SF), benchmarked against an unmodified binder and a commercial FEM. Two virgin asphalt binders from Egyptian refineries (Suez and Alex) were selected to capture source-dependent behavior. The novelty of this work lies in directly comparing organic and inorganic modifier classes under identical processing and characterization conditions, thereby providing mechanistic and transferable insights for the design of multifunctional, sustainable, and resilient asphalt binders.

## Research methodology

**Fig. 1:**
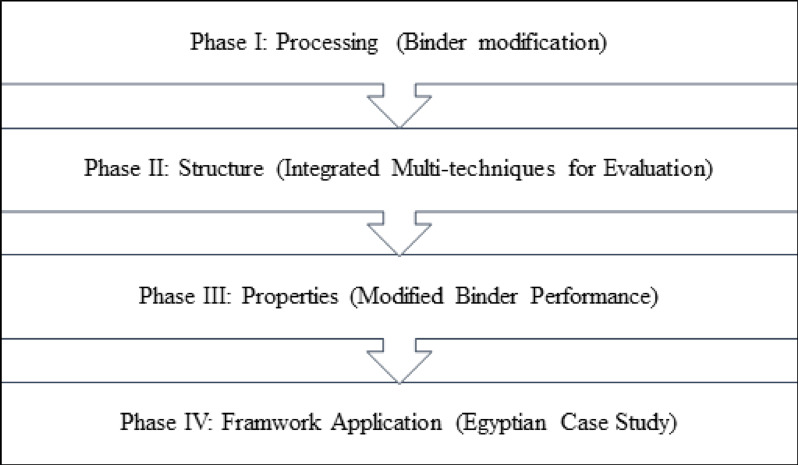
Research methodology.

The research methodology, illustrated in Fig. 1, follows a processing–structure–properties paradigm implemented through four interconnected phases. Phase I (Processing) focuses on binder modification using different organic and inorganic modifiers. Phase II (Structure) employs integrated multi-scale characterization techniques to capture chemical, thermal, and microstructural changes induced by modification and aging. Phase III (Properties) evaluates the engineering performance of the modified binders, including workability, constructability, and performance grading. Finally, Phase IV (Framework Application) integrates the findings into a unified comparative framework, demonstrated through an Egyptian case study. Although the experimental program is contextualized using two Egyptian binders, the study aims to extract mechanistic and comparative insights into modifier–binder interactions that are transferable across binder types, rather than to provide statistical global generalization.

### Binder processing (materials and binder modification)

Two penetration-grade 60/70 asphalt binders sourced from Egyptian refineries were used: Alexandria Oil Company (AOC) and Suez Oil Company (SOC), hereafter referred to as Alex and Suez, respectively. Six modifiers were investigated: crumb rubber (CR), low-density polyethylene (LDPE), a CR/LDPE blend, fly ash geopolymer (FAG), metakaolin–silica fume geopolymer (MK-SF G), and a commercial fiber elastomer modifier (FEM, VIATOP). All modifiers were incorporated using the wet process.

Table 1 summarizes the physical and chemical properties of all base binders and modifiers. Modifier dosages were not arbitrarily selected but were adopted from ranges reported in previous studies that identified near-optimal performance for each modifier type, rather than being re-optimized in the present study. For instance, LDPE and CR contents were selected based on the optimal ranges reported in^34,45,46^. Similarly, MK-SF G contents were chosen in accordance with^47,48^. Finally, the VIATOP dosage was adopted from^33^. This approach enables a fair cross-comparative evaluation of different modifier classes while maintaining practical relevance.

**Table 1:** Description and properties of the various materials.

Material	Description/properties													
Alex Asphalt Binder (Alex)	60/70 asphalt binder. Penetration depth at 25°C: 6.5 mm. Performance grade: PG 64-22. Softening point temperature: 51°C. Rotational viscosity at 135°C: 0.797 Pa.s.													
Suez Asphalt Binder (Suez)	60/70 asphalt binder. Penetration depth at 25°C: 7.5 mm. Performance grade: PG 58-22. Softening point temperature: 50.5°C. Rotational viscosity at 135°C: 0.539 Pa.s.													
LDPE	Second-grade plastic bags made of Low-Density Polyethylene (LDPE). Particle size: smaller than US Standard Sieve #50 and larger than Sieve #100. Chemical formula: C₂H₄. Density: 0.93 g/cm³. Melting point: 115°C. Thickness: 0.024 mm. Tensile strength: 10.2 MPa.													
CR	Recycled crumb rubber (CR) obtained from waste tires purchased from junk wholesalers in Egypt. Ground CR particles size is smaller than the US standard Sieve #50 and larger than Sieve #100.													
Geopolymer containing Metakaolin and Silica fumes (MK-SF G)	Generated in the laboratory (as it cannot be found locally) based upon research conducted by^29^ :The composition of MK-SF G is shown below:													
	Chemical component	MK		SF		NaOH		Na_2_SiO_3_		SiO_3:_Al_2_O_3_		Na_2_SiO_3:_NaOH		L:S
	Percent	50.2		12.2		12.5		25.1		3.7		2		0.6
Geopolymer containing Fly Ash (FA G)	Fly ash (FA) obtained from Sika Egypt for Construction Chemicals. Fly ash is used as an aluminosilicate source of material of Class F^47^. The composition of the FAG is shown below:													
	Chemical component		FA		NaOH		Na2SiO3		SiO3: Al2O3		Na2SiO3: NaOH		L:S	
	Percent		65		11.6		23.4		3.63		2		0.54	
FEM (VIATOP)	A commercially available additive. Made of a pelletized blend of natural cellulose fibers. Pelletized blend of 20% by weight cellulose fiber and 80% by weight functional additive. FEM density :1.2 g/cm3. FEM breakdown: 200 °C.													

#### Processing rationale and parameter selection

Processing parameters (modifier content, temperature, shear rate, and blending duration) were tailored to the physicochemical nature of each modifier, following validated procedures reported in the literature. Applying identical processing conditions to all modifiers would result in incomplete dispersion or thermal degradation for certain materials. Therefore, material-specific processing protocols were adopted to ensure effective incorporation, and resulting performance differences are interpreted in light of both material chemistry and processing requirements. A concise summary of key processing parameters is provided in Table 2.

**Table 2:** Summary of processing parameters for modified asphalt binders.

Modifier system	Modifier content (by wt. of binder)	Mixing temperature (°C)	Shear rate (rpm)	Mixing duration (min)	Processing rationale
LDPE	5%	163	2000	75	Temperature selected above LDPE melting point (115 °C) to ensure complete melting and dispersion without thermal degradation; parameters adopted from validated studies^45,46^.
Crumb Rubber (CR)	7%	163 (binder) + 175–200 (CR preheating)	2000	75	Elevated CR preheating activates rubber swelling and enhances interaction with asphalt maltene phase; widely reported as necessary for effective CR modification^45,46^
CR + LDPE	3% CR + 3% LDPE	163	2000	75	Combined protocol balances rubber swelling and polymer melting to promote synergistic dispersion while avoiding excessive viscosity increase.
Fly Ash Geopolymer (FAG)	12%	150	2000	120	Lower temperature limits premature geopolymer degradation while allowing sufficient interaction between the inorganic network and binder matrix^29,37^.
Metakaolin–Silica Fume Geopolymer (MK-SF G)	4%	150	2000	120	Optimized dosage and temperature selected to maximize aluminosilicate network formation without excessive stiffening^29,37^
Fiber Elastomer Modifier (FEM/VIATOP)	10%	170	350	35	Lower shear rate and moderate temperature prevent fiber damage and excessive viscosity increase; parameters follow manufacturer guidance and prior studies^33^.

Processing parameters were selected based on material-specific physicochemical requirements reported in previous validated studies. All modified binders were prepared following identical procedures for each modifier system to ensure repeatability, and resulting performance differences are interpreted in light of both material chemistry and processing conditions.

#### Polymer-modified binders

LDPE and CR particles passing US Sieve #50 and retained on Sieve #100 were blended with unaged binders using a high-shear mixer at 163 °C and 2000 rpm for 75 min. LDPE and CR were added at 5% and 7% by weight, respectively. CR particles were preheated to 175–200 °C before incorporation to activate rubber swelling and enhance interaction with the maltene phase. In contrast, LDPE was incorporated at a lower temperature once fully melted above its melting point (115 °C). This distinction reflects the fundamentally different incorporation mechanisms of elastomeric rubber versus thermoplastic polymer and is well supported by previous studies^45,46^.

For the hybrid system, 3% LDPE + 3% CR (by binder weight) were incorporated using the same procedure. Mixing temperatures and shear conditions were selected to ensure adequate dispersion while avoiding thermal degradation.

#### Geopolymer-modified binders

Metakaolin was synthesized by calcining kaolin at 650 °C for 8 h, producing a highly reactive aluminosilicate precursor. Geopolymers were prepared following the formulations reported in^29,37,48^. FAG and MK-SF G were incorporated into 500 g of virgin binder at 12% and 4% by weight, respectively, and shear mixed at 150 °C and 2000 rpm for 120 min.

#### FEM (VIATOP)-modified binders

The commercial fiber elastomer modifier (VIATOP) was incorporated at 10% by weight of binder and shear mixed at 170 °C and 350 rpm for 35 min, following^33^. Modifier contents were held constant across both binder sources to isolate source-dependent behavior. Binder-specific optimization is recognized as an important topic for future investigation.

#### Integrated multi-technique binder evaluation

**Fig. 2:**
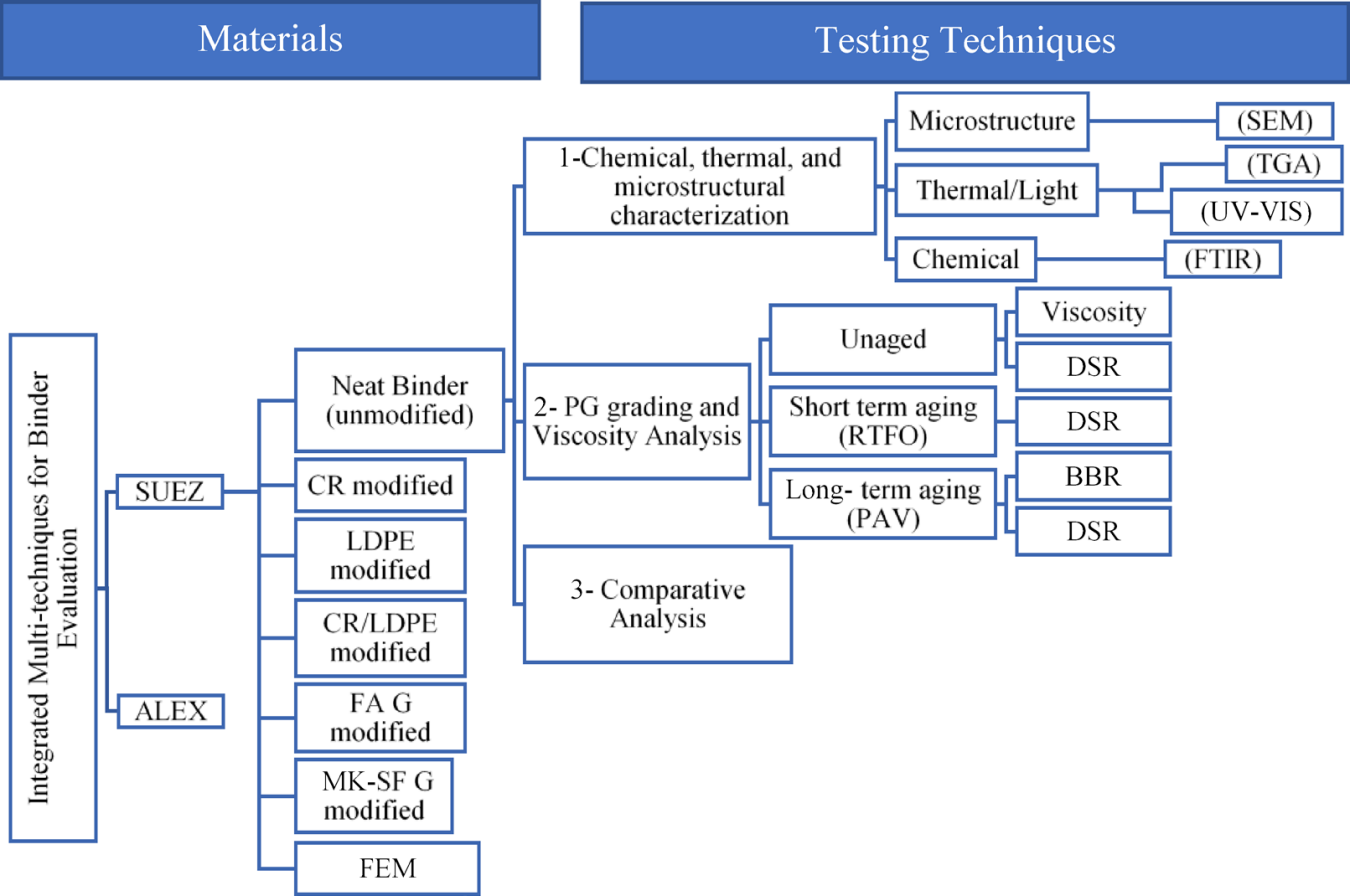
Framework structure (integrated multi-techniques for binder evaluation).

The integrated evaluation framework, shown in Fig. 2, combines chemical, thermal/light, microstructural, and rheological analyses to assess fourteen binder blends under three aging conditions: unaged, RTFO-aged (AASHTO T240), and PAV-aged (AASHTO R28). All modified binders were prepared following identical procedures to ensure repeatability. Rheological tests were conducted in duplicate, and reported values represent the average results. For SEM, FTIR, TGA, and UV–Vis analyses, multiple specimens were prepared, and representative results are presented based on consistent trends observed across samples.

#### FTIR analysis

FTIR testing was conducted in accordance with ASTM E1252-98 over the range 4000–400 cm⁻¹ at a resolution of 4 cm⁻¹. Samples were dissolved in the solvent before testing. Representative spectra are shown, and carbonyl (C = O) and sulfoxide (S = O) indices were used to assess oxidative aging.

#### SEM analysis

SEM analysis was performed using a Nikon JCM-6000Plus microscope. Samples were gold-coated (0.6 nm) and examined at 15 kV. All SEM images were acquired under identical accelerating voltage, magnification, and working distance, and multiple regions were examined to confirm microstructural consistency.

#### TGA analysis

TGA testing followed ASTM E1131-20, heating samples at 10 °C/min under nitrogen to 800 °C. Representative thermograms are shown, and mass-loss trends were used to evaluate thermal stability and aging sensitivity.

#### UV aging and UV–Vis characterization

UV aging was conducted in accordance with ASTM G154. Asphalt binder films (~ 0.5 mm thick) were cast on glass substrates using controlled spacers. Samples were exposed to UV radiation at 0.68 W/m² at 340 nm for 240 h under continuous exposure. After UV aging, films were recovered and analyzed using UV–Vis spectroscopy (ASTM E275/E275M) over the range 200–800 nm, with measurements recorded at 1 nm intervals. Representative spectra are shown.

#### Rheological testing

Rotational viscosity and PG grading were conducted following AASHTO T316 and T315 on unaged binders, while AASHTO PP42 and T313 were applied to PAV-aged samples. All rheological results represent the average of two replicates.

#### Comparative framework integration

Finally, results from all techniques were integrated to establish a unified comparative framework linking processing parameters, structural evolution, and engineering performance across modifier classes and binder sources. This framework forms the basis for the rational selection of sustainable modifiers and was demonstrated through the Egyptian case study.

## Results and discussion

### Binder properties: microstructural, chemical, and thermal

To evaluate the influence of different modifiers on asphalt binder behavior, particularly with respect to aging susceptibility, the chemical, thermal, light-induced, and microstructural characteristics of the modified binders were examined using a coordinated set of complementary analytical techniques, namely FTIR, TGA, UV–Vis spectroscopy, and SEM. SEM analysis was employed to qualitatively assess morphology, modifier dispersion, and microstructural stability, while FTIR, TGA, and UV–Vis analyses provided corresponding evidence of chemical transformation, thermal decomposition behavior, and photo-oxidative aging response. It is emphasized that the scientific value of the proposed framework lies not in the isolated use of individual techniques, but in their coordinated and comparative application, which enables the identification of consistent performance trends and modifier–binder interaction mechanisms that remain obscured when properties are evaluated independently. To ensure reliable cross-comparison, all SEM micrographs and corresponding FTIR, TGA, and UV–Vis results were generated and presented using consistent acquisition settings, scaling, and reference conditions wherever applicable.

### Microstructural evolution and aging response of modified asphalt binders

#### Effect of binder source and aging

**Fig. 3:**
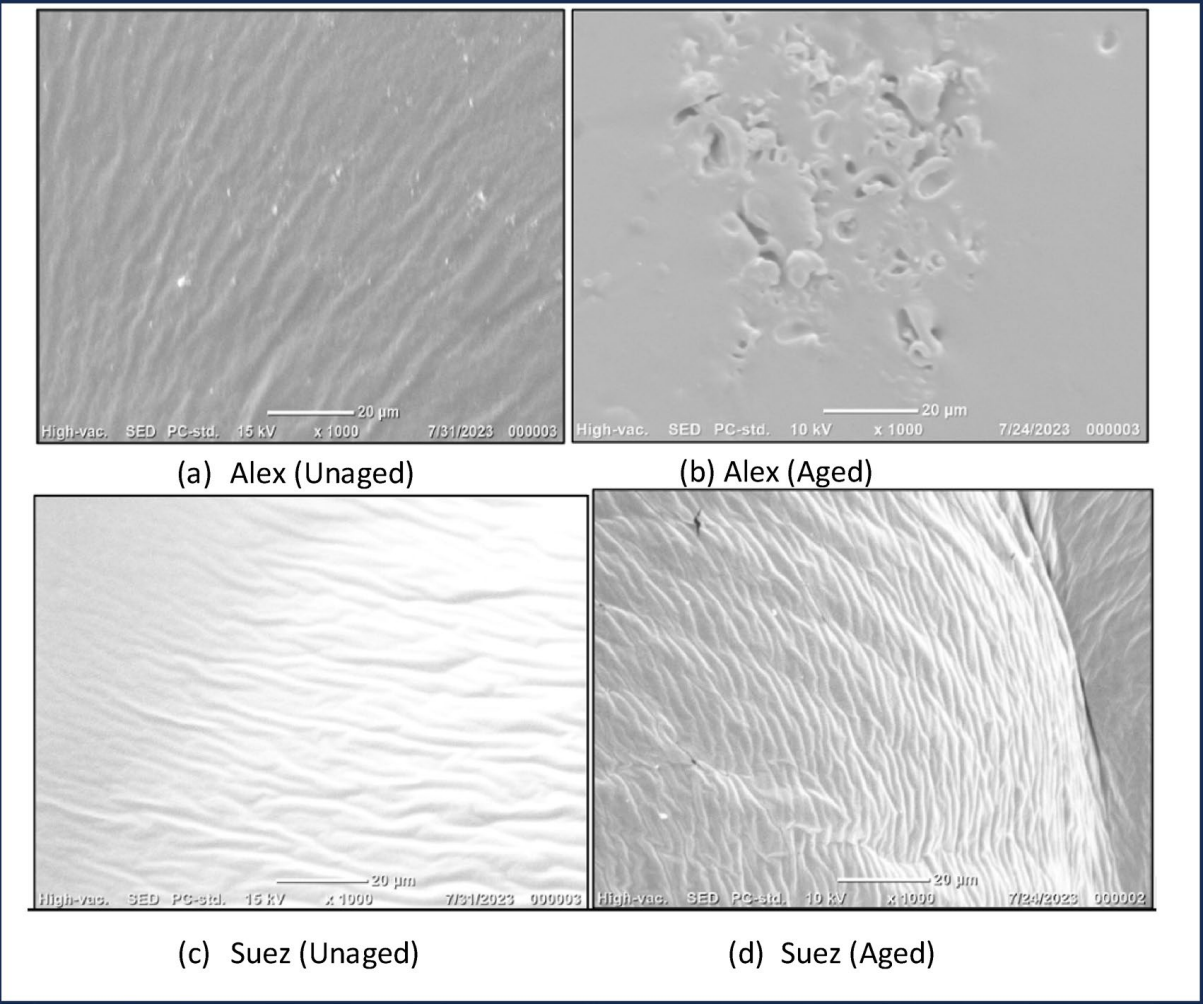
SEM microstructures of virgin asphalt binders illustrating source-dependent aging response: (**a**,**b**) Alex binder before and after aging, and (**c**,**d**) Suez binder before and after aging. The Alex binder exhibits pronounced microstructural degradation after aging, characterized by pore formation and surface discontinuities, whereas the Suez binder retains a more continuous and compact morphology, indicating higher intrinsic resistance to aging

SEM micrographs of the unaged Alex binder (Fig. 3a) display a heterogeneous surface morphology with a relatively coarse texture and localized bright features, reflecting compositional non-uniformity at the microscale. In contrast, the unaged Suez binder (Fig. 3c) exhibits a more continuous and homogeneous surface with fewer visible discontinuities, suggesting a more uniform microstructural organization. After aging, the Alex binder undergoes marked microstructural deterioration, evidenced by the development of surface porosity and irregular void-like features (Fig. 3b). Under the same aging conditions, the Suez binder shows only minor morphological changes and largely retains its compact and continuous structure (Fig. 3d). Although SEM provides qualitative insight, the consistent emergence of surface discontinuities in the aged Alex binder, relative to the limited changes observed in the Suez binder, indicates a higher susceptibility to aging-induced microstructural degradation. These source-dependent differences establish a critical baseline for evaluating the effectiveness of subsequent binder modifications.

#### Effect of waste-derived polymer modifiers

**Fig. 4:**
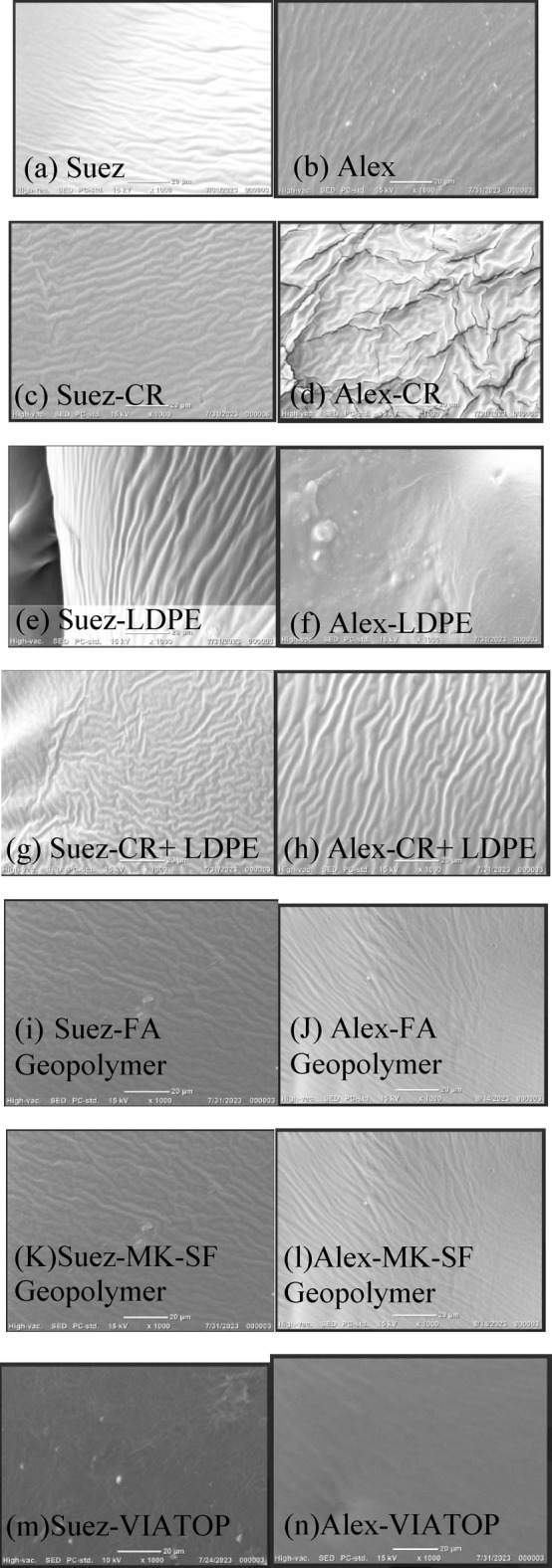
SEM microstructures of modified asphalt binders comparing the influence of modifier type and binder source on phase dispersion and microstructural continuity: (**a**–**b**) neat binders, (**c**–**d**) CR-modified, (**e**–**f**) LDPE-modified, (**g**–**h**) CR+LDPE-modified, (**i**–**j**) FA geopolymer-modified, (**k**–**l**) MK–SF geopolymer-modified, and (**m**–**n**) FEM (VIATOP)-modified binders (Suez vs. Alex). Geopolymer and CR-based modifications exhibit more homogeneous and continuous morphologies, whereas LDPE and VIATOP show increased heterogeneity and phase discontinuities, particularly in the Alex binder.

SEM micrographs of CR-modified binders (Fig. 4c,d) reveal interconnected, wavy microstructural features consistent with rubber swelling and partial incorporation within the asphalt matrix. The Suez–CR binder exhibits a more uniform distribution of these features than the Alex–CR system, indicating more effective rubber dispersion and improved microstructural continuity in the former. In contrast, LDPE-modified binders (Fig. 4e,f) display large, discrete bright domains characteristic of phase separation and limited compatibility with the asphalt matrix. This behavior is more pronounced in the Alex binder, underscoring the strong influence of base binder chemistry on LDPE dispersion. Similar dispersion limitations for LDPE-modified asphalt systems have been reported in the literature^34^. Notably, the hybrid CR/LDPE system (Fig. 4g,h) exhibits markedly improved dispersion relative to LDPE alone. The reduction of large isolated polymer domains and the emergence of a more continuous microstructural network suggest a synergistic interaction in which the presence of CR promotes improved LDPE distribution within the binder. While SEM observations are qualitative, this enhanced microstructural uniformity provides a consistent microstructural basis for the improved thermal, chemical, and rheological responses observed for the CR/LDPE blend at larger scales.

#### Effect of geopolymer and commercial modifiers

SEM micrographs of geopolymer-modified binders incorporating fly ash (FA Geopolymer) and metakaolin–silica fume (MK–SF Geopolymer) (Fig. 4i–l) exhibit smooth and relatively homogeneous surface morphologies with finely distributed inorganic phases. Among these systems, the MK–SF Geopolymer-modified binders display the least visible surface discontinuities, indicating a high degree of microstructural uniformity and effective dispersion of the geopolymer phase within the asphalt matrix.

In contrast, VIATOP-modified binders (Fig. 4m,n) show a less uniform morphology, characterized by localized bright domains and emerging surface irregularities. Although the unaged VIATOP-modified binders appear relatively homogeneous, these features suggest a comparatively lower microstructural stability, as discussed in the subsequent aging analyses.

#### Aging response of modified binders

**Fig. 5:**
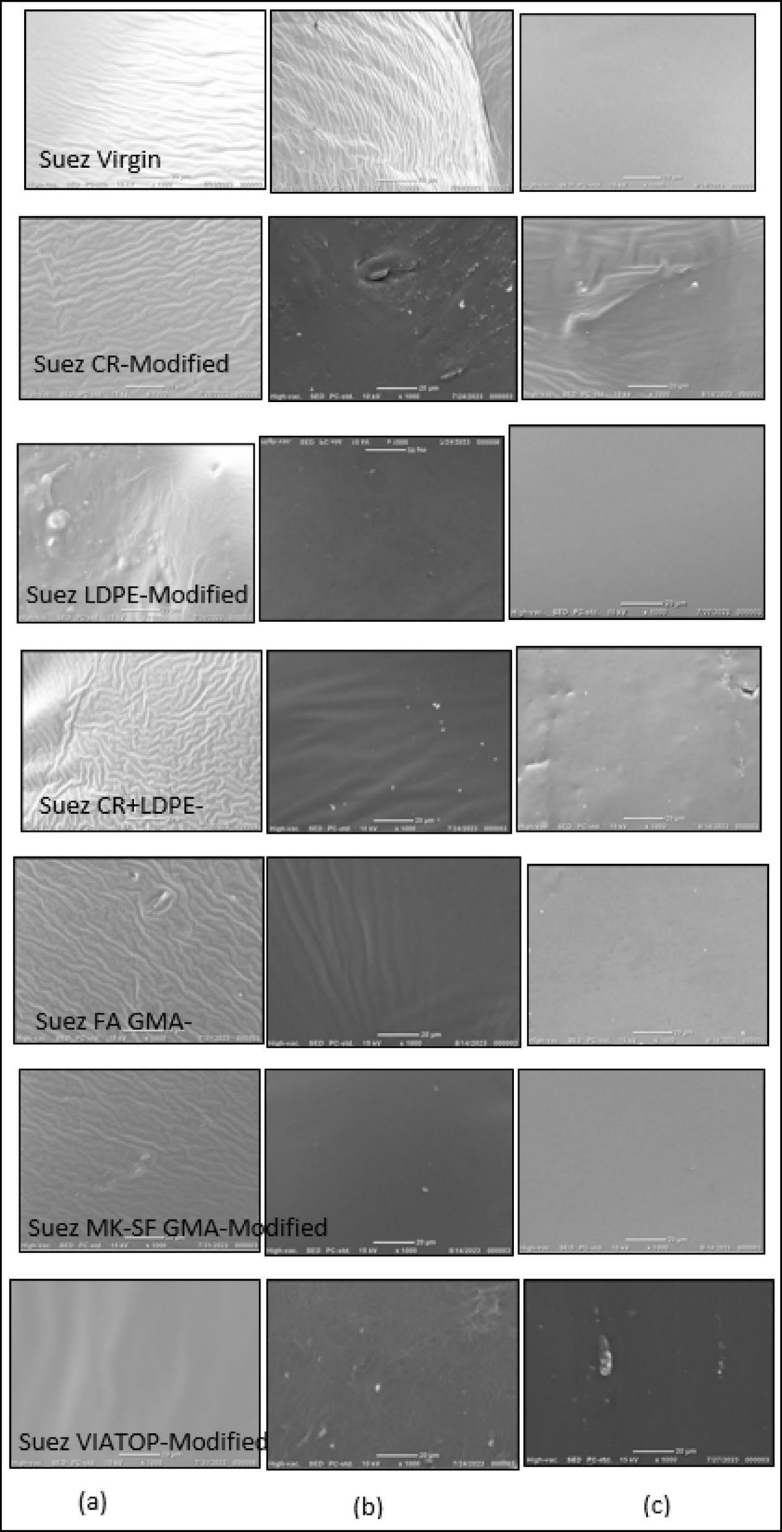
SEM microstructures of Suez-based asphalt binders illustrating the evolution of microstructural features with aging: (**a**) unaged, (**b**) RTFO-aged, and (**c**) PAV-aged conditions. Geopolymer- and CR-modified binders exhibit minimal microstructural degradation with aging, whereas LDPE- and VIATOP-modified binders show progressive surface heterogeneity and discontinuity, indicating reduced resistance to aging-induced microstructural deterioration.

The evolution of binder microstructure with aging was further examined using Suez-based binders modified with different additives under unaged, short-term aged (RTFO), and long-term aged (PAV) conditions (Fig. 5). Geopolymer-modified binders exhibit minimal morphological changes across aging stages, with limited development of surface discontinuities or pore-like features, indicating a comparatively high resistance to aging-induced microstructural degradation.

In contrast, VIATOP-modified binders show a progressive increase in microstructural heterogeneity with aging severity, manifested by the emergence and growth of localized bright domains after long-term aging (Fig. 5c). Although SEM does not permit direct chemical identification of these features, their systematic development with aging severity is consistent with increased phase instability and oxidation susceptibility, as independently supported by FTIR and TGA analyses discussed in the subsequent sections.

Based on the consistent qualitative trends observed across all aging stages, the relative resistance to microstructural aging can be ranked as:

geopolymer modifiers > CR ≈ CR/LDPE > LDPE > VIATOP.

While SEM provides qualitative insight, the agreement between this ranking and the corresponding chemical and thermal indicators reinforces the robustness of the multi-scale interpretation.

### FTIR and chemical aging indices: oxidation behavior and modifier effectiveness

Fourier Transform Infrared (FTIR) spectroscopy was employed to examine the evolution of functional groups and oxidative aging in modified asphalt binders^33^. To move beyond qualitative spectral inspection, oxidation was quantified using the Carbonyl Index (CI) and Sulfoxide Index (SI), which are widely accepted indicators of chemical aging severity.

**Fig. 6:**
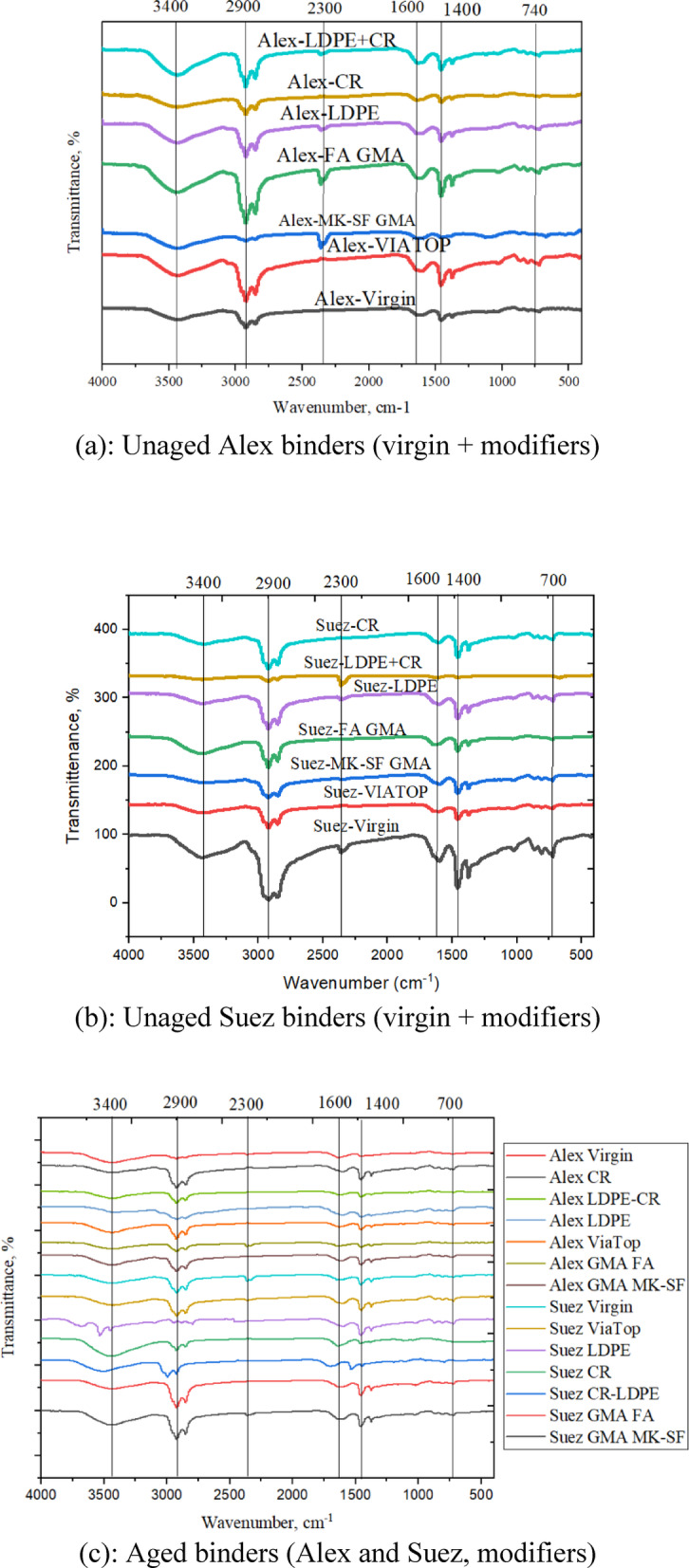
FTIR spectra of asphalt binders illustrating the influence of modifier type, binder source, and aging: (**a**) unaged Alex binders, (**b**) unaged Suez binders, and (**c**) aged binders after oxidative aging. Variations in the intensities of hydroxyl (~3400 cm⁻¹), aliphatic (~2900 cm⁻¹), aromatic (~1600 cm⁻¹), carbonyl (~1700 cm⁻¹), and sulfoxide (~1030 cm⁻¹) bands highlight source-dependent oxidation susceptibility and the effectiveness of different modifiers in mitigating chemical aging.

As shown in Fig. 6, both Alex and Suez binders exhibit characteristic absorption bands associated with hydroxyl groups (∼3400 cm⁻¹), aliphatic C–H stretching (∼2900 cm⁻¹), and aromatic C = C stretching (∼1600 cm⁻¹). The Alex binder consistently displays higher baseline absorbance across these regions, indicating a greater proportion of oxidation-prone constituents. This compositional distinction provides a chemical basis for the higher aging susceptibility of the Alex binder observed in subsequent analyses.

**Fig. 7:**
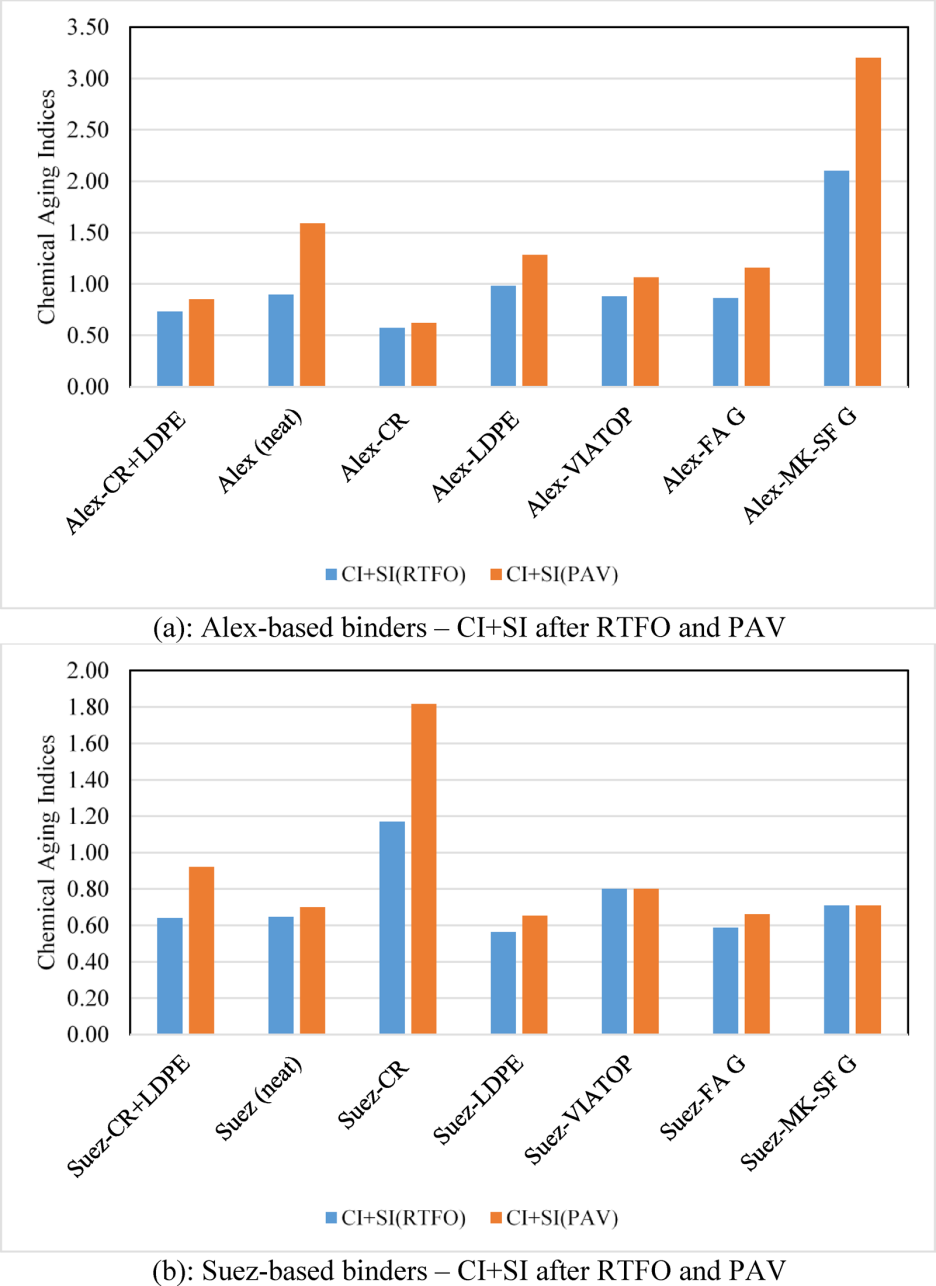
Combined carbonyl and sulfoxide indices (CI+SI) of modified asphalt binders after short-term (RTFO) and long-term (PAV) aging: (**a**) Alex-based binders and (**b**) Suez-based binders. Higher CI+SI values indicate increased oxidative aging. Geopolymer-modified binders exhibit the lowest aging indices, whereas LDPE- and VIATOP-modified binders show elevated oxidation, with stronger source dependency observed for the Alex binder

Aging-induced oxidation is reflected by the progressive growth of carbonyl (C = O, ∼1720 cm⁻¹) and sulfoxide (S = O, ∼1030 cm⁻¹) absorption bands following RTFO and PAV aging, as illustrated in Fig. 6c. To enable direct quantitative comparison across binder sources and modifier types, the combined CI + SI values were calculated. Figure 7 presents the CI + SI indices for fourteen binders after RTFO and PAV aging.

Across all binders, CI + SI values increase from RTFO to PAV aging, confirming progressive oxidation with increasing aging severity. However, the magnitude of this increase is strongly dependent on both binder source and modifier type. Unmodified binders exhibit pronounced CI + SI growth, with the Alex binder showing the largest increase after PAV aging, confirming its inherent chemical instability and high susceptibility to oxidative degradation.

Geopolymer modifiers exhibit contrasting behavior depending on the base binder chemistry. Fly ash (FA) geopolymer-modified binders consistently show reduced CI + SI values for both Alex and Suez sources, indicating effective suppression of oxidation. In contrast, the MK–SF geopolymer leads to the highest CI + SI value for the Alex binder after PAV aging, suggesting that the highly reactive Alex base binder dominates the aging response and overrides the protective effect of this modifier. This finding highlights the critical role of base binder chemistry in governing modifier effectiveness.

Waste-derived polymer modifiers display pronounced source-dependent trends. For the Suez binder, CR modification results in a noticeable increase in CI + SI after PAV aging, indicating enhanced oxidative product formation despite the favorable rubber dispersion observed in SEM. This response can be rationalized by considering the intrinsic chemical stability of the Suez binder. Because the Suez binder initially contains a lower concentration of oxidation-prone species, the relative introduction of rubber-related unsaturated components and reactive interfaces may proportionally increase measurable oxidation indices during severe aging. In other words, the chemically stable base matrix of Suez allows CR-related oxidative reactions to become more detectable in the CI + SI metrics.

In contrast, the Alex binder, which exhibits higher baseline oxidation susceptibility, experiences partial mitigation when modified with CR. In this case, rubber swelling and absorption of lighter fractions may dilute or redistribute reactive species, leading to a relative reduction in measurable oxidation compared to the highly unstable unmodified Alex binder. These findings demonstrate that modifier effectiveness is governed not only by dispersion quality but by the interaction between modifier chemistry and the intrinsic oxidative balance of the base binder.

LDPE-modified binders show moderate CI + SI reductions relative to unmodified binders for both sources, suggesting a limited but consistent protective effect against oxidation. The hybrid CR/LDPE system generally exhibits intermediate behavior, reflecting competing effects between rubber-induced absorption and polymer phase instability.

The commercial fiber elastomer modifier (VIATOP) exhibits strong source dependency. VIATOP increases CI + SI values for the Suez binder, indicating reduced aging resistance, while its effect on the Alex binder is comparatively neutral. This divergence further confirms that modifier performance cannot be evaluated independently of base binder chemistry.

Based on CI + SI values after PAV aging (Fig. 7), the relative resistance to chemical oxidation can be ranked as:

FA geopolymer > LDPE ≈ CR/LDPE > CR > FEM (VATOP) > unmodified binder, with the MK–SF geopolymer exhibiting beneficial behavior only when paired with a chemically stable base binder.

Overall, the integration of FTIR spectral evolution (Fig. 6) with quantitative CI + SI indices (Fig. 7) establishes a clear chemical basis for the observed microstructural and thermal trends. These results confirm that aging resistance is governed by the interaction between modifier chemistry and base binder composition rather than modifier type alone.

### Thermogravimetric analysis (TGA): thermal stability and performance implications

**Fig. 8:**
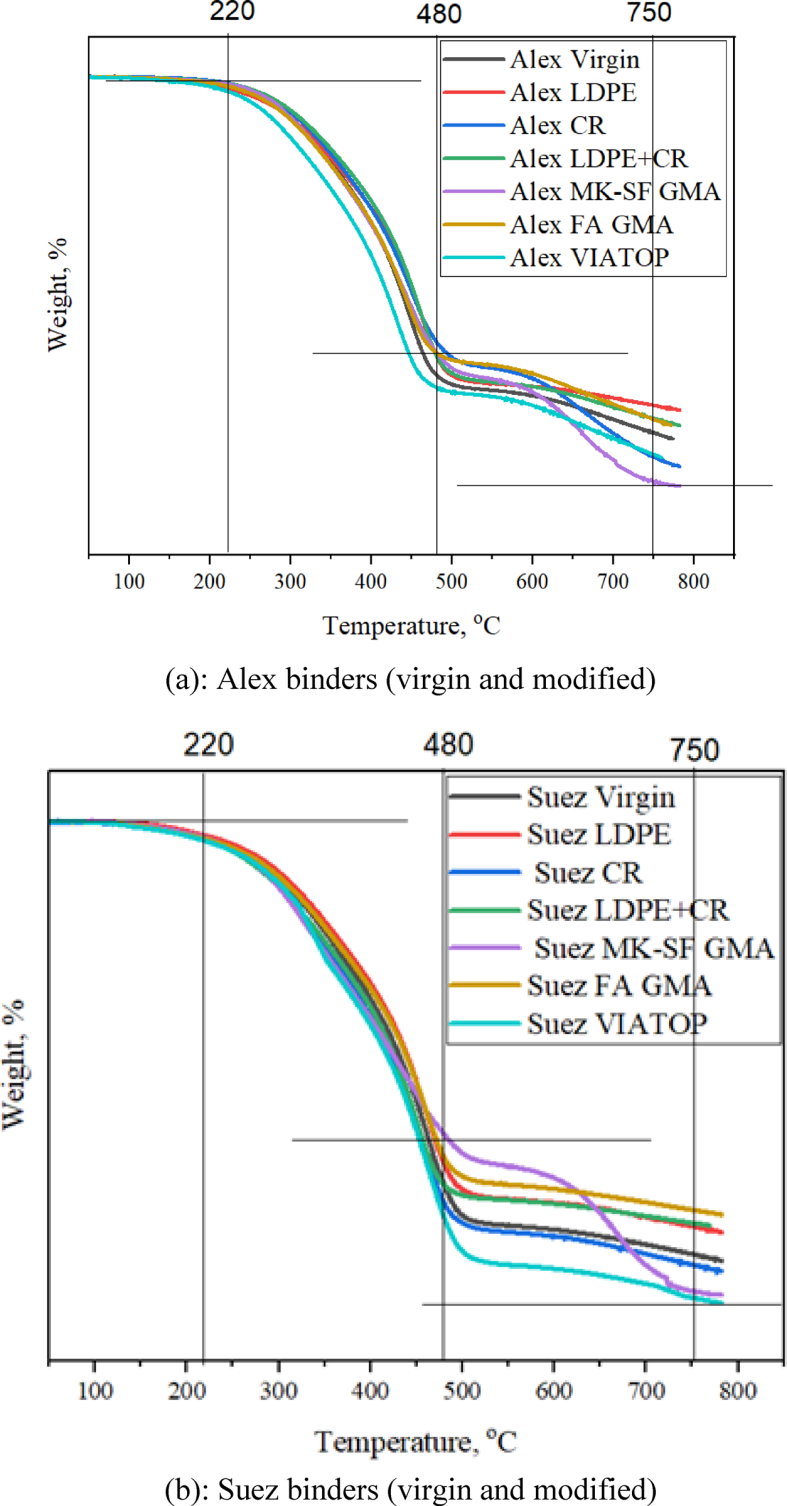
Thermogravimetric analysis (TGA) of modified asphalt binders from Alex and Suez sources. Thermograms illustrate source- and modifier-dependent differences in thermal decomposition behavior, including onset temperature, degradation rate, and residual mass. Geopolymer- and CR-modified binders exhibit enhanced thermal stability, while LDPE and VIATOP show accelerated mass loss at elevated temperatures, highlighting the strong influence of modifier chemistry and base binder composition on thermal resistance.

Thermogravimetric analysis (TGA) was employed to evaluate the thermal stability and decomposition behavior of modified asphalt binders and to elucidate the influence of different modifiers on high-temperature durability^33,46^. Representative thermograms for Alex- and Suez-based binders are presented in Fig. 8.

Across all formulations, the TGA curves exhibit two dominant mass-loss stages. The first stage, occurring between approximately 220 and 480 °C, corresponds to volatilization and thermal decomposition of organic constituents, including maltenes and polymeric modifiers. The second stage, spanning roughly 480 to 750 °C, is associated with further degradation of heavier fractions and the evolution of inorganic or char-like residues. These temperature ranges are consistent with those reported for conventional and modified asphalt binders.

Quantitatively, the binders differ in onset decomposition temperature, rate of mass loss, and residual mass at elevated temperatures, which collectively reflect their thermal resistance. Virgin binders exhibit the earliest onset of major decomposition, typically initiating near 220–230 °C, and retain the lowest residual mass beyond 750 °C, indicating limited inherent thermal stability.

CR-modified binders show a modest increase in decomposition onset temperature (approximately 5–10 °C) relative to the virgin binders, together with slightly higher residual mass. This behavior reflects the thermally stable, crosslinked rubber structure, which retards volatilization of lighter fractions and delays degradation. These thermal trends are consistent with SEM observations of improved microstructural continuity and FTIR results indicating moderated oxidation.

In contrast, LDPE-modified binders exhibit slightly lower onset temperatures and steeper mass-loss rates within the primary decomposition region, particularly for the Alex binder. This response is attributed to the relatively lower thermal resistance of LDPE and its limited compatibility with the asphalt matrix, as evidenced by phase separation in SEM images and elevated oxidation indices from FTIR analysis. The hybrid CR+LDPE system displays intermediate behavior, indicating partial compensation between rubber-induced stabilization and polymer-induced vulnerability.

Geopolymer-modified binders (FA geopolymer and MK–SF geopolymer) demonstrate the highest thermal stability among all investigated systems. As shown in Fig. 8, these binders exhibit a clear rightward shift in decomposition onset (approximately 10–20 °C) and retain significantly higher residual mass at 750–800 °C compared to both virgin and polymer-modified binders. This enhanced thermal resistance is attributed to the formation of a rigid inorganic geopolymer network that restricts molecular mobility and suppresses volatile release. The persistence of higher residual mass is consistent with FTIR-derived evidence of reduced oxidation and SEM observations of stable, homogeneous microstructures.

VIATOP-modified binders, in contrast, show an earlier onset of decomposition and reduced residual mass, indicating limited thermal reinforcement. These results corroborate FTIR findings of increased oxidation severity (higher CI + SI values) and SEM observations of aging-induced phase instability, particularly for the Alex binder.

Based on combined TGA indicators, namely the onset temperature of major decomposition, the rate of mass loss in the primary degradation region, and the residual mass retained at high temperature, the relative thermal stability of the investigated modifiers can be qualitatively ranked as:

FA geopolymer ≥ MK–SF geopolymer > CR ≈ CR+LDPE > LDPE > VIATOP > unmodified binder.

From a performance perspective, the TGA results indicate how effectively each modifier enhances binder durability under high-temperature production and service conditions. A higher onset decomposition temperature signifies improved resistance to volatilization and oxidative hardening during mixing and long-term exposure, while greater residual mass reflects the presence of thermally stable structural networks that delay molecular breakdown. Conversely, early decomposition and rapid mass loss accelerate stiffness growth and embrittlement, increasing cracking susceptibility. Accordingly, geopolymer- and CR-modified binders exhibit superior thermal resilience and durability, whereas LDPE- and VIATOP-modified systems provide limited thermal stabilization, particularly when applied to chemically unstable base binders.

### UV–Vis spectroscopy: photochemical aging behavior and cross-scale interpretation

UV–Vis spectroscopy was employed to evaluate the susceptibility of both Alex and Suez asphalt binders to UV-induced photochemical aging after modification. Changes in UV–Vis absorbance are associated with the formation of aromatic and conjugated structures during photo-oxidation, which contribute to binder hardening and increased aging sensitivity.

**Fig. 9:**
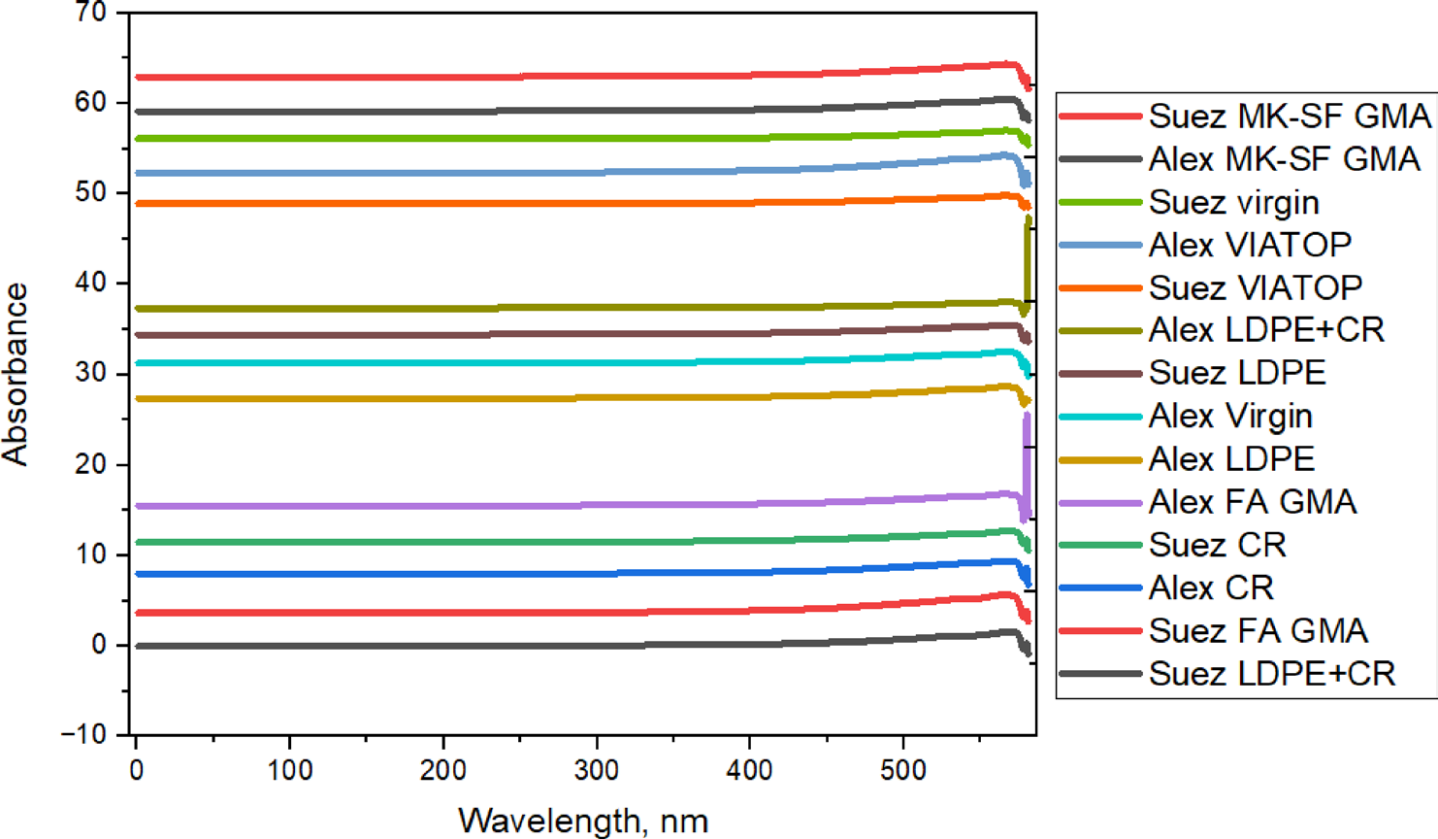
UV–Vis spectra of modified asphalt binders after UV aging, comparing the relative absorbance response of Alex and Suez binders as a function of modifier type. Lower absorbance levels indicate reduced formation of UV-induced aromatic and oxidized species, reflecting enhanced resistance to photochemical aging. Geopolymer- and CR-modified binders exhibit consistently lower absorbance compared to LDPE- and VIATOP-modified systems, with performance strongly dependent on base binder chemistry.

For comparative evaluation, a semi-quantitative absorbance ratio (AR) was used, defined as the ratio between the average absorbance in the aromatic/conjugated region (300–400 nm) and that in the aliphatic-dominated region (200–300 nm), calculated from the spectra shown in Fig. 9. Higher AR values indicate a greater relative contribution of aromatic/asphaltic structures and more advanced UV-induced aging.

As shown in Fig. 9, Alex-based binders consistently exhibit higher absorbance levels and higher AR values than their Suez counterparts, regardless of modifier type. This confirms a stronger susceptibility of the Alex binder to photochemical aging, in agreement with FTIR-derived oxidation indices (CI + SI), SEM observations of increased microstructural disruption, and TGA evidence of reduced thermal stability.

For both binder sources, CR-modified binders display lower absorbance levels and reduced AR values compared to the corresponding neat binders, indicating effective mitigation of UV-induced aromatic structure formation. This effect is more pronounced in the Suez binder, suggesting that the protective role of CR is enhanced when combined with a chemically stable base binder. These trends are consistent with SEM observations of improved microstructural continuity and FTIR results showing reduced oxidation growth.

In contrast, LDPE-modified binders show absorbance levels comparable to or slightly higher than those of the neat binders, indicating limited resistance to UV-induced aging. This behavior is observed for both Alex and Suez binders and aligns with SEM evidence of poor polymer dispersion, FTIR oxidation indices, and reduced thermal stability from TGA analysis.

Geopolymer-modified binders (FA geopolymer and MK–SF geopolymer) exhibit among the lowest absorbance levels and AR values within each binder source, particularly for the Suez binder. This indicates strong inhibition of UV-induced formation of aromatic and conjugated structures. The dense inorganic geopolymer network likely restricts UV penetration and suppresses photo-oxidative reactions. These UV–Vis results directly corroborate the low CI + SI values, stable SEM morphologies, and superior thermal stability observed in TGA analysis.

VIATOP-modified binders exhibit absorbance trends similar to those of LDPE-modified systems, indicating limited protection against photochemical aging. This behavior is consistent across both binder sources and aligns with FTIR evidence of increased oxidation and TGA observations of reduced thermal resistance.

The hybrid CR+LDPE system shows intermediate absorbance levels, lower than LDPE alone but generally higher than CR-modified binders. This indicates that CR partially compensates for the limited UV resistance of LDPE, resulting in moderated photochemical aging rather than full suppression. This intermediate behavior mirrors trends observed consistently across SEM, FTIR, CI + SI, and TGA analyses.

Based on absorbance ratio trends extracted from Fig. 9, the relative resistance to UV-induced aging can be qualitatively ranked as:

Geopolymer modifiers > CR > CR+LDPE > LDPE ≈ VIATOP > unmodified binder, with Suez-based binders consistently outperforming Alex-based binders. These results confirm that UV-aging resistance is governed by the combined influence of modifier chemistry and base binder composition, reinforcing the multi-scale comparative framework proposed in this study.

### Rheological properties: workability and performance grading

To assess the practical applicability of the modified asphalt binders in pavement construction, rheological characterization was conducted focusing on workability (viscosity at 135 °C) and high- and low-temperature performance grading (PG). These parameters provide engineering-scale validation of the microstructural, chemical, thermal, and photochemical trends discussed in previous sections. All rheological measurements reported in this study represent the average of two independent replicates, conducted under identical testing conditions.

**Fig. 10:**
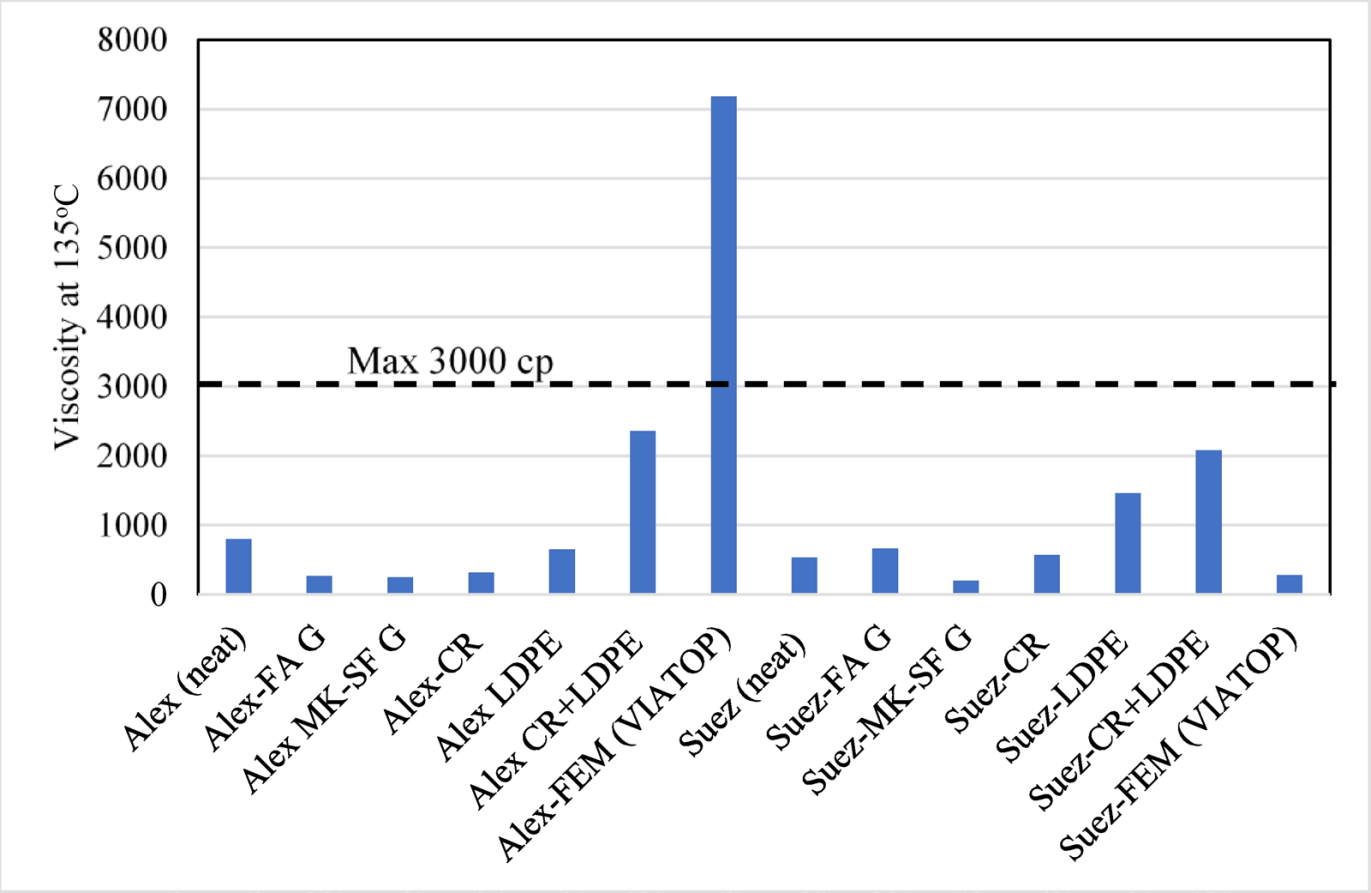
Rotational viscosity of neat and modified asphalt binders measured at 135 °C, illustrating the influence of modifier type and binder source on workability.

Figure 10 presents the viscosity of all binders measured at 135 °C. Except for the Alex VIATOP-modified binder, all samples satisfy the Superpave workability criterion of **≤** 3000 cP, indicating adequate pumpability and constructability. The Alex VIATOP binder exhibits a viscosity exceeding 7000 cP, substantially surpassing the specification limit. In contrast, the corresponding Suez VIATOP binder shows a viscosity of approximately 300 cP, well within acceptable limits. This pronounced source-dependent behavior highlights the dominant influence of base binder chemistry on rheological response and is consistent with earlier observations of inferior thermal and oxidative stability of the Alex binder.

Geopolymer-modified binders (FA geopolymer and MK-SF geopolymer) from both sources exhibit low viscosities (≈ 200–500 cP), reflecting minimal impact on workability. CR-modified binders show a slight viscosity increase relative to the neat Suez binder, while LDPE and CR+LDPE systems produce more pronounced increases, yet remain within acceptable limits. These viscosity trends align with SEM and FTIR results, where geopolymer modifiers showed minimal structural disruption, while polymer-based modifiers induced greater matrix stiffening.

The dashed line indicates the Superpave workability threshold (3000 cP). Most modified binders satisfy pumpability requirements, while VIATOP modification in the Alex binder exceeds the viscosity limit, highlighting strong source–modifier dependency and potential constructability constraints.

The performance grading results summarized in Table 3 further illustrate the effect of modifier type and binder source. Although the neat Alex binder exhibits a higher nominal PG (PG64-22) than the Suez binder (PG58-22), its true continuous PG reveals inferior overall durability when considered alongside chemical aging indices and thermal stability results. It is noted that PG determination follows Superpave specification limits based on threshold compliance of rheological parameters rather than statistical inference, and therefore PG shifts reflect engineering performance classification rather than variability-driven trends.

For Alex-based binders, LDPE, CR+LDPE, and VIATOP modifications result in the largest increase in true high-temperature PG, reaching PG 73.5–77.8, while CR modification produces a moderate increase (PG 67.5). Geopolymer modifications yield smaller PG shifts (PG ≈ 66), consistent with their role as stabilizing rather than stiffening agents.

For Suez-based binders, the highest true PG is achieved with VIATOP (PG 72.5), followed by LDPE (PG 71.2) and CR+LDPE (PG 68.9). CR modification provides a moderate improvement (PG 63.1), while geopolymer-modified binders again show the smallest PG increase. These results confirm that polymer-based modifiers primarily enhance high-temperature stiffness, whereas geopolymer modifiers preferentially improve aging resistance and stability without excessive stiffening.

Importantly, when rheological results are interpreted alongside SEM, FTIR (CI + SI), TGA, and UV–Vis analyses, a consistent multi-scale picture emerges: modifiers that strongly increase PG and viscosity (LDPE, VIATOP) tend to exhibit poorer aging resistance, while geopolymer and CR-based systems provide balanced performance by improving durability with acceptable workability and moderate stiffness enhancement.

While long-term rheological aging measurements were conducted as part of performance grade determination, rheological testing in this study is intended to provide engineering-scale context rather than a detailed aging analysis, which is addressed through complementary chemical, thermal, and microstructural evaluations.

**Table 3:** Table 3 Performance grades of modified binders.

Asphalt binder (source and type)	Nominal performance grade (PG)*	True (continuous) performance grade (PG)
Alex (neat)	PG64-22	PG 65.31–27.96
Alex-FA G	PG64-28	PG 66.90–28.01
Alex-MK-SF G	PG64-22	PG 66.02–27.88
Alex-CR	PG64-22	PG 67.50 −22.05
Alex-LDPE	PG70-22	PG 73.50–22.00
Alex-CR+LDPE	PG76-22	PG 77.76–22.02
Alex-VIATOP	PG64-28	PG 67.13 −28.00
Suez (neat)	PG58-22	PG 58.49–22.04
Suez-FA G	PG58-22	PG 62.07–22.05
Suez-MK-SF G	PG58-22	PG 58.53–22.03
Suez-CR	PG58-22	PG 63.14–22.39
Suez-LDPE	PG70-28	PG 71.15–28.21
Suez-CR+LDPE	PG64-22	PG 68.86–22.02
Suez-VIATOP	PG70-16	PG 72.51–21.51

*PG values represent the average of two replicate measurements.

 Table4 consolidates the outcomes of the multi-scale evaluation by ranking each modifier across microstructural, chemical, thermal, photochemical, and rheological performance indicators. The table highlights that modifiers exhibiting consistent improvements across all scales, most notably geopolymer systems and crumb rubber, provide balanced enhancements in durability, aging resistance, and constructability. In contrast, certain modifiers, particularly LDPE and VIATOP, display apparent contradictions between rheological performance grading and physicochemical stability. While these modifiers significantly increase the high-temperature PG through binder stiffening, SEM, FTIR, CI + SI, TGA, and UV–Vis results reveal inferior dispersion, accelerated oxidation, and reduced thermal and photochemical stability. This discrepancy indicates that PG improvement alone reflects short-term stiffness enhancement rather than long-term material resilience. For LDPE and VIATOP, the increase in PG is primarily driven by physical hardening and phase effects, which can elevate workability resistance but simultaneously promote aging-related degradation. The integrated assessment summarized in Table 4, therefore, underscores the necessity of multi-scale evaluation, demonstrating that sustainable binder design must balance rheological gains with chemical and microstructural stability to ensure durable pavement performance.

**Table 4:** Comparative multi-scale ranking of asphalt modifiers across microstructural, chemical, thermal, photochemical, and rheological evaluations.

Modifier	SEM (microstructural stability)	FTIR (oxidation resistance)	CI+SI (chemical aging)	TGA (thermal stability)	UV–Vis (UV aging resistance)	Rheology (workability & PG balance)	Overall performance
FA geopolymer	Excellent	Excellent	Excellent	Excellent	Excellent	Moderate	Excellent
MK–SF geopolymer	Excellent	Good–Excellent*	Moderate–Poor*	Excellent	Good	Moderate	Good
Crumb rubber (CR)	Good	Good	Good	Good	Good	Moderate	Good
CR + LDPE	Good	Moderate	Moderate	Moderate	Moderate	Good	Moderate
LDPE	Poor–Moderate	Poor	Poor–Moderate	Poor	Poor	Excellent (PG increase)	Poor–Moderate
VIATOP (FEM)	Poor (after aging)	Poor	Poor	Poor	Poor	Poor (Alex) / Good (Suez)	Poor
Unmodified binder	Poor	Poor	Poor	Poor	Poor	Moderate	Poor

* Performance strongly dependent on base binder chemistry (Alex vs. Suez).

### Framework application (Egyptian case study)

Climatic performance requirements for Egyptian pavements were evaluated using the Long-Term Pavement Performance (LTPP)-based framework reported in^49^, considering both low-reliability (50%) and high-reliability (98%) design levels. For low-reliability projects, required base PG values range from PG52-10 to PG64-10 depending on climatic zone, whereas high-reliability projects require PG64-10 to PG76-10.

**Fig. 11:**
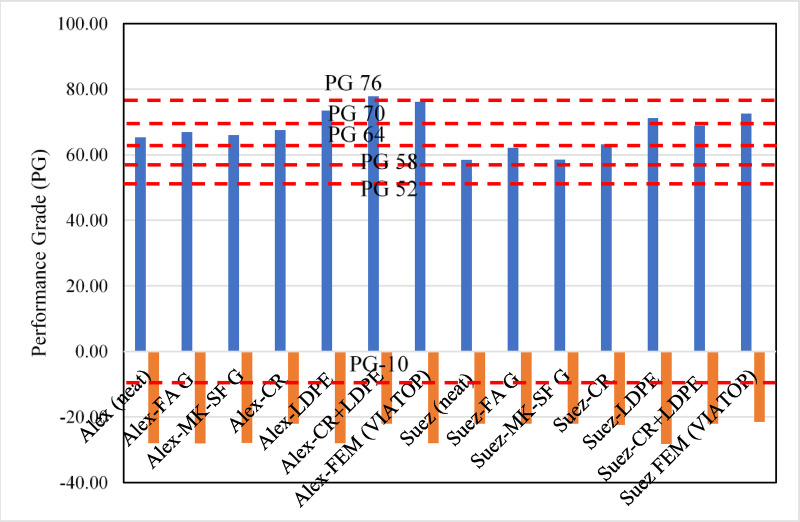
Comparison of true (continuous) performance grades of modified asphalt binders relative to Egyptian nominal PG limits. Continuous PG values were determined from rheological test temperatures and plotted against nominal Egyptian PG class boundaries (shown as dashed lines) for contextual comparison. The results highlight source- and modifier-dependent stiffness enhancement, with polymer-based modifiers producing the largest high-temperature PG increases.

As depicted in Fig. 11, the neat Suez binder (PG 58.49–22.04) satisfies only part of the low-reliability requirements and fails to meet high-reliability, high-temperature demands. The neat Alex binder (PG 65.31–27.96) meets low-reliability criteria and approaches certain high-reliability zones but remains marginal for extreme conditions. These findings confirm that base binder selection alone is insufficient for high-reliability climatic performance.

Modifier incorporation significantly increased the high-temperature true PG values, particularly for LDPE, CR+LDPE, and VIATOP systems, thereby extending applicability toward higher climatic zones. However, although performance grading (PG) remains the standardized and widely accepted specification tool for binder selection, it is fundamentally based on rheological thresholds and therefore primarily reflects stiffness-related limits rather than the underlying chemical and microstructural mechanisms governing durability. The present results show that reliance on PG improvement alone may obscure trade-offs between stiffness enhancement and long-term aging resistance.

While polymer-based modifiers (LDPE and VIATOP) produced the largest PG shifts, they simultaneously exhibited inferior oxidation resistance, thermal stability, and UV durability. In contrast, geopolymer- and CR-based systems delivered moderate PG improvement while substantially enhancing chemical and thermal aging resistance. This distinction highlights that modifiers that maximize high-temperature PG do not necessarily provide the most durable solution under severe climatic exposure.

Importantly, most sustainable modified binders satisfy low-reliability climatic requirements and meet the low-temperature PG criteria across reliability levels. However, only limited formulations approach the highest high-reliability high-temperature thresholds (PG76-10), indicating that stiffness enhancement alone is insufficient for extreme climatic design without concurrent durability optimization.

This case study demonstrates the practical value of the proposed processing–structure–properties framework. By integrating rheological grading with microstructural (SEM), chemical (FTIR, CI + SI), thermal (TGA), and photochemical (UV–Vis) analyses, the framework enables durability-informed modifier selection and prevents over-reliance on PG shifts as the sole indicator of performance. Within this integrated perspective, geopolymer and CR systems emerge as the most reliable candidates for sustainable and climate-resilient pavement applications, particularly where long-term durability is critical.

### Conclusions and recommendations

This study developed and validated a unified multi-scale comparative framework for evaluating sustainable asphalt modifiers by integrating microstructural (SEM), chemical (FTIR and CI + SI), thermal and photochemical (TGA and UV–Vis), and rheological (viscosity and PG) analyses across twelve modified binders derived from two Egyptian base asphalt sources. The framework enabled direct comparison of modifier performance under consistent processing and testing conditions, addressing limitations of prior single-property studies.

Quantitatively, geopolymer-modified binders (FA and MK–SF) exhibited the most consistent improvements across scales, achieving the lowest chemical aging indices (CI + SI), the highest decomposition onset temperatures (≈ 10–20 °C increase), minimal UV-induced absorbance growth, and stable microstructures, while maintaining workable viscosities **(**< 500 cP at 135 °C) and moderate PG increases **(** ≈ + 2–4 °C). Crumb rubber (CR) provided balanced performance, yielding moderate PG enhancement **(** ≈ + 4–5 °C), improved thermal stability, and reduced oxidative and UV aging without exceeding workability limits.

In contrast, LDPE and VIATOP produced the largest increases in high-temperature PG (up to + 10–13 °C) but simultaneously showed higher oxidation indices, earlier thermal decomposition, UV-induced aromatic growth, and microstructural phase separation.

These results demonstrate that PG enhancement alone reflects short-term stiffness gain rather than long-term durability. The observed LDPE incompatibility, which contrasts with some previous studies, is mechanistically attributed to poor chemical affinity between non-polar polyethylene chains and polar asphalt fractions, leading to phase segregation, limited oxidative shielding, and accelerated aging, effects that become pronounced when binder chemistry (e.g., Alex source) is unfavorable. Similarly, VIATOP’s fiber-based stiffening improves PG but promotes localized stress concentration and oxidation under prolonged thermal and UV exposure. Moreover, the contrasting behavior of VIATOP across binder sources highlights that fiber-based modifiers primarily provide physical reinforcement and are highly sensitive to the intrinsic chemical and thermal stability of the base binder.

The CR+LDPE hybrid system exhibited intermediate behavior, where rubber partially mitigated LDPE phase instability, resulting in improved dispersion and moderated aging, though not matching the durability of geopolymer or CR-only systems. Across all modifiers, binder source played a decisive role: despite a higher nominal PG, the Alex binder consistently exhibited higher oxidation, poorer thermal stability, and inferior aging resistance than the Suez binder, confirming that base binder chemistry governs the effectiveness of modification strategies.

Design-Oriented Recommendations.

1. Primary choice – Geopolymers (FA, MK–SF**)**: Recommended where long-term aging resistance, thermal stability, and UV durability are critical, particularly in hot or high-radiation climates.
2. Balanced performance – CR: Suitable when moderate PG enhancement is required without compromising durability or workability.
3. Synergistic blends – CR+LDPE: Promising for applications requiring combined stiffness and flexibility, provided further optimization is performed.
4. Conditional use – LDPE and VIATOP: Should be applied cautiously; PG gains must be weighed against durability losses, and compatibility enhancement strategies are necessary before large-scale use.
5. Binder selection: Base binder chemistry must be considered a primary design variable; stable sources (e.g., Suez) yield more reliable outcomes.
6. Future work: Long-term field validation and molecular-scale modeling are recommended to refine predictive design rules for modifier–binder compatibility.

Overall, the proposed framework delivers quantitative, transferable design guidance for selecting sustainable asphalt modifiers, emphasizing that durable pavement performance requires balanced improvements across chemical, microstructural, thermal, and rheological scales, not stiffness enhancement alone.

## Data Availability

All data supporting the findings of this study are available within the paper.
