# Correction: A comparative evaluation of sustainable asphalt binder modifiers for enhanced performance

**DOI:** 10.1038/s41598-026-57790-x

**Published:** 2026-06-12

**Authors:** Maram Saudy, Minas Guirguis, Sherif EL-Badawy, Mohamed AbouZeid, Tarek Madkour

**Affiliations:** 1 The American University in Cairo, P.O. Box 74, New Cairo, 11835 Cairo Egypt; 2 Faculty of Engineering, Mansoura University, Mansoura, 35516 Egypt

*Correction to: Scientific Reports* 10.1038/s41598-026-46495-w, published online 13 April 2026

The original version of this Article contained an error in the name of author Sherif El-Badawy which was incorrectly given as Sherif ELBadawy.

The original Article has been corrected.

